# Renaissance of base deficit for the initial assessment of trauma patients: a base deficit-based classification for hypovolemic shock developed on data from 16,305 patients derived from the TraumaRegister DGU^®^

**DOI:** 10.1186/cc12555

**Published:** 2013-03-06

**Authors:** Manuel Mutschler, Ulrike Nienaber, Thomas Brockamp, Arasch Wafaisade, Tobias Fabian, Thomas Paffrath, Bertil Bouillon, Marc Maegele, the TraumaRegister DGU

**Affiliations:** 1 Department of Trauma and Orthopedic Surgery, Cologne-Merheim Medical Center (CMMC), University of Witten/Herdecke, Ostmerheimerstr. 200, D-51109 Cologne, Germany; 2 Institute for Research in Operative Medicine (IFOM), University of Witten/Herdecke, Ostmerheimerstr. 200, D-51109 Cologne, Germany; 3 Academy for Trauma Surgery, Luisenstr. 58/59, D-10117 Berlin, Germany; 4 Working Group on Polytrauma of the German Society for Trauma Surgery (DGU), Luisenstr. 58/59, D-10117 Berlin, Germany

## Abstract

**Introduction:**

The recognition and management of hypovolemic shock still remain an important task during initial trauma assessment. Recently, we have questioned the validity of the Advanced Trauma Life Support (ATLS) classification of hypovolemic shock by demonstrating that the suggested combination of heart rate, systolic blood pressure and Glasgow Coma Scale displays substantial deficits in reflecting clinical reality. The aim of this study was to introduce and validate a new classification of hypovolemic shock based upon base deficit (BD) at emergency department (ED) arrival.

**Methods:**

Between 2002 and 2010, 16,305 patients were retrieved from the TraumaRegister DGU^® ^database, classified into four strata of worsening BD [class I (BD ≤ 2 mmol/l), class II (BD > 2.0 to 6.0 mmol/l), class III (BD > 6.0 to 10 mmol/l) and class IV (BD > 10 mmol/l)] and assessed for demographics, injury characteristics, transfusion requirements and fluid resuscitation. This new BD-based classification was validated to the current ATLS classification of hypovolemic shock.

**Results:**

With worsening of BD, injury severity score (ISS) increased in a step-wise pattern from 19.1 (± 11.9) in class I to 36.7 (± 17.6) in class IV, while mortality increased in parallel from 7.4% to 51.5%. Decreasing hemoglobin and prothrombin ratios as well as the amount of transfusions and fluid resuscitation paralleled the increasing frequency of hypovolemic shock within the four classes. The number of blood units transfused increased from 1.5 (± 5.9) in class I patients to 20.3 (± 27.3) in class IV patients. Massive transfusion rates increased from 5% in class I to 52% in class IV. The new introduced BD-based classification of hypovolemic shock discriminated transfusion requirements, massive transfusion and mortality rates significantly better compared to the conventional ATLS classification of hypovolemic shock (p < 0.001).

**Conclusions:**

BD may be superior to the current ATLS classification of hypovolemic shock in identifying the presence of hypovolemic shock and in risk stratifying patients in need of early blood product transfusion.

## Introduction

The early recognition and management of hypovolemic shock in multiply injured patients are still among the most challenging tasks in the acute assessment and treatment of trauma patients. For the initial evaluation of circulatory depletion, the American College of Surgeons has defined in its training program 'Advanced Trauma Life Support' (ATLS) four classes of hypovolemic shock. This classification is based upon an estimated blood loss in percent together with corresponding vital signs [1,2]. For each class, ATLS allocates therapeutic recommendations (for example, the administration of intravenous fluids and blood products) [1]. Recently, the clinically validity of the ATLS classification of hypovolemic shock has been questioned by two analyses independently from each other on two large-scale trauma databases: the TARN (Trauma Audit and Research Network) registry and the TraumaRegister DGU^®^, which had consisted of more than 140,000 trauma patients. According to both analyses, ATLS seems (a) to overestimate the degree of tachycardia associated with hypotension and (b) to underestimate mental disability in the presence of hypovolemic shock [3-5].

These observations and conclusions prompted us to develop an alternative approach for the early assessment of hypovolemic shock in the emergency department (ED). Several studies have already identified worsening base deficit (BD) as an indicator for increased transfusion requirement [6,7]. Furthermore, BD has been associated with increased mortality, intensive care unit (ICU) and in-hospital lengths of stay, and a higher incidence of shock-related complications such as acute respiratory distress syndrome, renal failure, hemocoagulative disorders, and multiorgan failure (MOF) [6-9]. Monitoring of BD has also been suggested as an indicator and monitoring parameter for the success of resuscitation efforts [7,10,11]. In times of point-of-care testing (POCT), BD can be assessed in a fast and easy manner and therefore is available within minutes after admission to the ED. The aim of this study was to introduce and validate a four-class BD-based classification of hypovolemic shock on datasets of severely injured patients derived from the TraumaRegister DGU^® ^database.

## Materials and methods

### The TraumaRegister DGU^®^

The TraumaRegister DGU^® ^was founded in 1993 and details have been published *in extenso *elsewhere [3,12]. To date, datasets from approximately 70,000 patients from more than 450 hospitals have been entered into the database. The TraumaRegister DGU^® ^captures all severe trauma patients, who either are admitted to the hospital via the ED with subsequent ICU/intermediate care (ICU/IMC) care or reach the hospital with vital signs and die prior to ICU/IMC admission. It was approved by the review board of the German Trauma Society (DGU) and is in compliance with the institutional requirements of its members.

### Data analyses

In the present study, datasets of multiply injured patients entered into the TraumaRegister DGU^® ^between 2002 and 2010 were analyzed. Inclusion criteria were age of at least 16 years, primary admission, and complete datasets for BD upon admission blood gas analysis as well as for systolic blood pressure (SBP), heart rate (HR), and Glasgow Coma Scale (GCS) score to rebuild the ATLS classification of hypovolemic shock for validation.

### Characterization of the four classes of hypovolemic shock based upon base deficit at emergency department admission

According to Davis and colleagues [6], four different classes of shock were defined and analyzed. Class I ('no shock') was defined by a BD of not more than 2 mmol/L, class II ('mild shock') by a BD of more than 2.0 to 6.0 mmol/L, class III ('moderate shock') by a BD of more than 6.0 to 10.0 mmol/L, and class IV ('severe shock') by a BD of more than 10 mmol/L. Each patient was allocated to the corresponding shock class I to IV according to BD upon ED arrival. Vital signs (for example, HR, SBP, and GCS score) were assessed as present upon ED arrival and at the scene of the accident. Shock index (SI), defined by the ratio of HR to SBP, was calculated for both time points. Further assessments included demographics and injury patterns as well as therapeutic interventions such as administration of blood products, intravenous fluids, and vasopressors. Massive transfusion (MT) was defined by the administration of at least 10 blood products between ED and ICU admission. Coagulopathy was defined by a Quick's value of not more than 70%, which is equivalent to an international normalized ratio of approximately 1.3 [13,14].

### Validation of the new base deficit-based classification to the current ATLS classification of hypovolemic shock

For the validation of the new BD-based classification to the current ATLS classification of hypovolemic shock, the latter was interpreted as previously described [3]. Briefly, SBP, HR, and GCS score were assessed to allocate the patients into the respective ATLS groups of hypovolemic shock but with some minor modifications [3]. As stated above, allocation of patients into the respective classes of hypovolemic shock was limited if a combination of all three parameters was applied. Therefore, in the present analysis, we allocated each patient into the respective shock class I to IV by the vital sign (HR, SBP, or GCS score) that matches the criteria of the highest shock class. If patients had been intubated and mechanically ventilated prior to ED admission, the GCS score at the scene of injury was considered. Patients were classified according to their BD at ED admission and according to the criteria suggested by ATLS. Transfusion requirements as well as mortality rates within the four groups were compared.

### Statistical methods

Data are presented as means ± standard deviations for continuous variables or percentages for categorical variables. GCS scores are presented as medians and interquartile ranges. For continuous variables, normal distribution was excluded by using the Shapiro-Wilk test. To detect differences between the four groups of worsening BD, a Kruskal-Wallis test was performed. A Mann-Whitney *U *test on pairwise comparisons was performed in case of a significant overall difference. Categorical variables were analyzed accordingly with the chi-square test. For all statistical analyses, a probability of less than 0.05 was considered to be statistically significant. All data were analyzed by using IBM SPSS 19 (IBM Corporation, Chicago, IL, USA).

## Results

### Characterization of the four classes of hypovolemic shock based upon base deficit at emergency department admission

In total, 16,305 patients were identified from the TraumaRegister DGU^® ^for further analysis. General demographics and detailed information on injury severity, trauma mechanism, RISC (Revised Injury Severity Classification) prognosis, and outcome for the four classes of hypovolemic shock based upon BD at ED admission are shown in Table 1. Worsening of BD category was associated with increased injury severity and both increased morbidity and mortality. Consequently, ICU and overall in-hospital lengths of stay as well as times on ventilator were prolonged with worsening of BD category. Table 2 summarizes vital signs for the four classes of shock at the scene and upon ED admission. A significant increase in SI was observed through the groups I to IV. HR seemed unaltered within the four groups, and interestingly no group displayed a relevant tachycardia at all. A substantial hypotension with a mean SBP of 87 ± 45 mm Hg was observed in patients with a BD of more than 10 mmol/L (class IV) only. GCS scores decreased from a median of 14 (3 to 15) in class I patients to 3 (3 to 3) in class IV patients, whereas the percentage of patients intubated and mechanically ventilated at the scene increased from 40.2% (class I) to 83.4% (class IV), respectively. Furthermore, hemoglobin levels dropped from 12.8 ± 2.4 g/dL (class I) to 9.1 ± 3.3 g/dL (class IV), and platelet counts declined substantially throughout the classes I to IV (Table 3). Coagulopathy, defined by a Quick's value of not more than 70%, was found in patients with a BD of more than 6 mmol/L (classes III and IV).

**Table 1:** Patients classified by base deficit (classes I to IV): demographics, injury mechanism, severity of injury, and outcome

	Class I	Class II	Class III	Class IV
	
	BD ≤ 2.0 (no shock)	BD > 2.0 to 6.0 (mild)	BD > 6.0 to 10.0 (moderate)	BD > 10.0 (severe)
Demographics				

Total number (percentage)	7,583 (46.5)	5,831 (35.8)	1,999 (12.3)	892 (5.5)
Male, number (percentage)	5,622 (74.7)	4,184 (72.3)	1,382 (69.6)	607 (68.4)
Mean age (SD), years	46 (20.2)	43.8 (19.7)	44.4 (19.5)	45.8 (19.7)
Blunt trauma, number (percentage)	7,088 (96)	5,436 (94.4)	1,869 (94.1)	816 (92.3)

Injury severity				

Mean ISS (SD), points	19.1 (11.9)	24.0 (13.3)	29.5 (16)	36.7 (17.6)
Mean NISS (SD), points	24.2 (15)	29.9 (16.1)	35.5 (17.7)	42.9 (18.5)
Mean RISC score (SD), points	10.3 (18.1)	14.4 (22.4)	24.4 (28.6)	53.3 (35.3)
AIS head ≥ 3 points, number (percentage)	3,065 (40.4)	2,711 (46.5)	1,039 (52)	526 (59)
AIS thorax ≥ 3 points, number (percentage)	2,826 (37.3)	2,811 (48.2)	1,131 (56.6)	577 (64.7)
AIS abdomen ≥ 3 points, number (percentage)	819 (10.8)	939 (16.1)	520 (26.0)	296 (33.2)
AIS pelvis/extremities ≥ 3 points, number (percentage)	1,956 (25.8)	2,071 (35.5)	846 (42.3)	419 (47.0)

Outcome				

Mortality, number (percentage)	564 (7.4)	721 (12.4)	478 (23.9)	459 (51.5)
Mean hospital LOS (SD), days	18,3 (19.2)	23.6 (25.2)	24.7 (27)	20.1 (31.2)
Mean ICU LOS (SD), days	7.8 (10.7)	11.3 (13.3)	13.9 (15.3)	12.8 (18.0)
Mean ventilator days (SD)	4.8 (8.9)	7.7 (11.5)	9.9 (12.8)	10.1 (15.5)
Multiple organ failure, number (percentage)	807 (12.2)	1,064 (20.2)	516 (29.4)	294 (43.3)
Sepsis, number (percentage)	400 (6.0)	566 (10.5)	295 (16.3)	126 (18.0)

Cohort consisted of 16,305 patients. *P *< 0.001 for all parameters. AIS, abbreviated injury scale; BD, base deficit; ICU, intensive care unit; ISS, injury severity score; LOS, length of stay; NISS, new injury severity score; RISC, Revised Injury Severity Classification; SD, standard deviation.

**Table 2:** Patients classified by base deficit (classes I to IV): traditional vital signs as presented at emergency department admission and at the scene

	Class I	Class II	Class III	Class IV
	
	BD ≤ 2.0 (no shock)	BD > 2.0 to 6.0 (mild)	BD > 6.0 to 10.0 (moderate)	BD > 10.0 (severe)
Vital signs				

SBP at the scene, mm Hg	129.8 (28.9)	120.7 (32.2)	108.6 (35.1)	87.2 (45.4)
SBP at ED, mm Hg	132.6 (26.3)	124.6 (28.0)	112.7 (30.7)	94.8 (40.4)
HR at the scene, beats per minute	90.9 (19.3)	93.9 (22.6)	98.3 (27)	93.6 (41)
HR at ED, beats per minute	86.3 (17.8)	89.8 (20.3)	95.9 (22.5)	97.2 (32.4)
SI at the scene, beats per minute	0.74 (0.26)	0.83 (0.34)	0.98 (0.47)	1.17 (0.55)
SI at ED, beats per minute	0.68 (0.22)	0.76 (0.27)	0.93 (0.41)	1.17 (0.69)
Median GCS score at the scene (IQR), points	14 (10-15)	13 (6-15)	10 (3-15)	4 (3-12)
Median GCS score at ED (IQR), points	14 (3-15)	3 (3-15)	3 (3-11)	3 (3-3)
Intubation rate, number (percentage)	2,732 (40.2)	3,319 (60.5)	1,417 (73.9)	724 (83.4)

Values are presented as mean (standard deviation) unless indicated otherwise. Cohort consisted of 16,305 patients. *P *< 0.001 for all parameters. BD, base deficit; ED, emergency department; GCS, Glasgow Coma Scale; HR, heart rate; IQR, interquartile range; SBP, systolic blood pressure; SI, shock index.

**Table 3:** Patients classified by base deficit (classes I to IV): laboratory findings

	Class I	Class II	Class III	Class IV
	**BD ≤ 2.0** **(no shock)**	**BD > 2.0 to 6.0** **(mild)**	**BD > 6.0 to 10.0** **(moderate)**	**BD > 10.0** **(severe)**

Laboratory findings				

Hemoglobin, g/dL	12.8 (2.4)	11.8 (2.6)	10.6 (2.9)	9.1 (3.3)
Thrombocytes, tsd/μL	215 (74)	208 (77)	193 (81)	171 (82)
Quick's value, percentage	85.9 (19.7)	79.7 (21.6)	69.6 (24)	55.5 (26.1)
aPTT, seconds	29.8 (9.2)	32.1 (13.7)	39.0 (23)	69.5 (41.1)
Lactate, mmol/L	2.5 (4.3)	3.4 (5.4)	5.1 (8.4)	9.7 (14)

Values are presented as mean (standard deviation). Cohort consisted of 16,305 patients. *P *< 0.001 for all parameters. aPTT, activated partial thromboplastin time; BD, base deficit.

**Figure 1:**
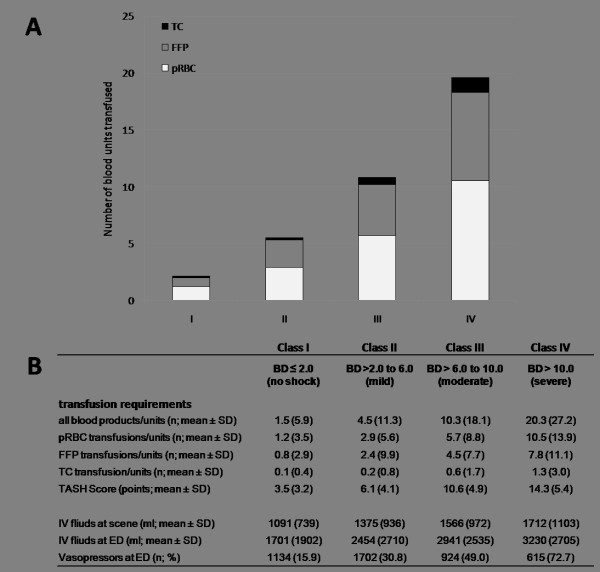
**Hemostatic and fluid resuscitation in patients classified by base deficit (BD) into classes I to IV**. **(a) **Total amounts of packed red blood cells (pRBCs), fresh frozen plasma (FFP), and thrombocyte concentrate (TC) transfused. **(b) **Transfusion requirements and fluid resuscitation (*n *= 16,305; *P *< 0.001). ED, emergency department; IV fluids, intravenous fluids; SD, standard deviation; TASH, Trauma-Associated Severe Hemorrhage.

An increase in BD category was associated with a progressively stepwise increasing number of blood products administered (Figure 1). On average, the number of blood units transfused increased from 1.5 ± 5.9 units in class I patients to 20.3 ± 27.3 units in class IV patients. Packed red blood cells were transfused most frequently, followed by fresh frozen plasma and platelet concentrates (Figure 1a). Simultaneously, observed and predicted transfusion requirements were concordant, as the number of blood products transfused paralleled increased TASH (Trauma-Associated Severe Hemorrhage) scores. Similarly, both fluid administration and the use of vasopressors increased through groups I to IV (Figure 1b).

### Validation of the new base deficit-based classification to the current ATLS classification of hypovolemic shock

**Figure 2:**
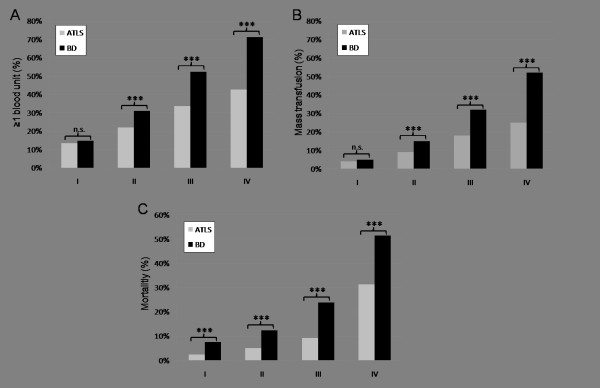
**Transfusion requirements and mortality in patients classified according to either admission base deficit (BD) or the ATLS classification of hypovolemic shock**. **(a) **Percentage of patients with at least one blood product. **(b) **Percentage of patients with massive transfusion, defined as at least 10 blood units until intensive care unit (ICU) admission. **(c) **Mortality (percentage). ***P < 0.001; *n *= 16,305. ATLS, Advanced Trauma Life Support; n.s., not significant.

When the two approaches to classify the extent of hypovolemic shock upon ED admission were compared, the new BD-based classification displayed a higher accuracy for discriminating the need for early blood products than the current ATLS classification of hypovolemic shock (Figure 2). Through groups II to IV, the percentage of patients who had received at least 1 blood unit during early ED resuscitation was significantly higher compared with patients classified according to ATLS (Figure 2a). A similar pattern was noted for the frequency of MTs (Figure 2b). If patients were classified by BD, MT rates increased from 5% in class I (BD of not more than 2 mmol/L) to 52% in class IV (BD of more than 10 mmol/L). In contrast, when patients were classified according to ATLS, 4% of group I and only 25% of group IV patients received MT until ICU admission (Figure 2b). Furthermore, BD distinguished more precisely between patients at risk of dying than the current ATLS classification of hypovolemic shock (Figure 2c). If classified by BD, 7.4% of class I and 51.5% of class IV patients, on average, died during in-hospital stay. In contrast, patients classified according to ATLS showed mortality rates of 2% in class I and 31% in class IV patients.

## Discussion

The aim of this study was to introduce and validate a new BD-based classification of hypovolemic shock for the initial assessment of trauma patients. This analysis was conducted on a cohort of not less than 16,305 severely injured patients derived from the TraumaRegister DGU^® ^database.

The early assessment of hypovolemic shock and the prediction of transfusion requirements in multiply injured patients are still among the most challenging tasks in the initial management of trauma patients. One approach comprises the initial evaluation of vital signs as suggested by ATLS in its classification of hypovolemic shock by using combinations of HR, SBP, and GCS score. However, recent analyses on data of multiply injured patients derived from the TraumaRegister DGU^® ^and the TARN database indicated that the current ATLS classification of hypovolemic shock displays substantial deficits in allocating trauma patients into the corresponding classes [3,4]. Furthermore, the role of vital signs alone in the initial assessment of hypovolemic shock is still debated [3,15-18]. Paladino and colleagues [19] recently assessed the additional use of metabolic parameters (for example, BD as a sensitive indicator of blood loss by measuring tissue perfusion) to traditional triage vital signs to distinguish major from minor trauma. In their retrospective single-center analysis, abnormal vital signs alone had a sensitivity of 40.9% for identifying major injury, but when abnormal metabolic parameters were added, the detection of major trauma increased significantly to a sensitivity of 76.4% [19].

In the present study, we propose a new classification based upon BD, a parameter that indicates the presence of hypovolemic shock and identifies patients who are at risk to require blood product transfusions. In times of POCT, BD is available within minutes after ED admission. As early as 2005, Rixen and Siegel [9] suggested the evaluation of BD as a more useful approach to quantify the extent of hypovolemic shock than the estimation of blood loss, the extent of volume resuscitation, or vital signs such as HR and SBP. Additionally, these authors proclaimed that BD may be superior to the measurement of lactate levels.

The diagnostic use and prognostic value of BD are well documented. Out of 10 clinical and 20 laboratory parameters assessed, changes of BD have been proven to be the best predictor of blood volume change in a canine model of hemorrhagic shock [20]. On the basis of 1,810 multiply injured trauma patients derived from the TraumaRegister DGU^® ^database, potential predictors for transfusion requirements, including BD and lactate, have been identified via logistic regression. Seven variables could be identified to independently predict MT: gender (male), SBP, HR, hemoglobin, relevant injuries to the abdomen and extremities (Abbreviated Injury Scale score of at least 3), and BD, but not lactate [21,22]. Furthermore, our group has recently compared six scoring systems to predict the risk of ongoing hemorrhage and MT, including the TASH, Prince of Wales Hospital/Rainer (PWH/Rainer), Larson, Vandromme, Schreiber, and ABC (assessment of blood consumption) scores. The TASH and PWH/Rainer scores showed the highest overall accuracy in predicting ongoing hemorrhage and MT. Interestingly, both scores include BD as a laboratory surrogate for hypoperfusion. In contrast, only one scoring system (that is, the Vandromme score) comprises lactate [23]. Similarly, several mortality scores (for example, the Emergency Trauma Score (EMTRAS) [24] and BIG score [25]) use BD as the laboratory surrogate for shock. In the present study, worsening BD paralleled worsening lactate. However, the use of Ringer's lactate in the initial fluid resuscitation as well as the presence of ketoacidosis in patients with diabetes may influence lactate levels and can falsify the initial assessment [9,26]. The present study did not intend to address the question of whether BD or lactate may be superior in risk-stratifying trauma patients, and therefore this question remains unanswered. However, the data derived from the TraumaRegister DGU^® ^database suggest that BD may be more accurate in detecting shock and blood loss as compared with lactate. Therefore, the proposed classification here is based on BD upon ED admission.

The present investigation revealed that increasing BD category reflected injury severity as demonstrated by an increasing injury severity score (ISS), new injury severity score (NISS), and RISC score and the incidence of MOF and sepsis. All of them are important factors influencing mortality and outcome of trauma patients. In our analysis, mortality rates rose from 7.4% to 51.5% with altered BD values. These observations are consistent with those of previous studies reporting an association between admission BD and mortality [6,7,10,11]. In a univariate logistic model, admission BD has been proven to be one of the best predictors for mortality, and a BD level of 6 mmol/L was identified as an important cutoff point for mortality [7,11]. Also, in pediatric and older trauma populations, BD has been shown to be an important indicator for injury severity and mortality [27-30]. Interestingly, the use of alcohol and drugs did not impair the predictive accuracy of admission BD with respect to trauma outcome [31].

In the present analysis, BD correlated with transfusion requirements, both in the overall amount of transfused blood units and in the percentage of patients who required any blood transfusion (≥ 1 blood unit). Furthermore, worsening BD paralleled increasing risk of ongoing hemorrhage as reflected by increasing TASH scores. The mean amount of blood products administered increased from 1.5 ± 5.9 to 20.3 ± 27.2 units with worsening BD category. These findings are consistent with those of a previous analysis demonstrating that worsening of BD was associated with an increased need for blood product transfusions [6,7,32]. Through the groups I to IV, the increasing amounts of intravenous fluids and vasopressors administered indicate the presence of hemodynamic instability and validated the results previously reported by Rixen and colleagues [7]. Laboratory findings such as decreases in hemoglobin levels and platelet counts and an impaired coagulation as reflected by a Quick's value of less than 70% were further interpreted as evidence for hypovolemic instability. Given these results, BD indicates the presence of hypovolemic shock related to hemostatic resuscitation need, transfusion requirements, laboratory findings, and mortality.

To the best of our knowledge, there is no gold standard to assess the presence of hypovolemic shock and to trigger therapeutic interventions. Thus, there is no option yet to test our novel approach against a gold standard. Therefore, the authors have decided to test against the current ATLS classification of hypovolemic shock given that this approach has been widely implemented in daily clinical routine as a standard protocol of care and for the initial assessment and treatment in trauma centers. Both the percentage of patients who had received at least one blood product and MTs were increased throughout the groups I to IV in both classifications. However, transfusion requirements were significantly higher when patients were classified by BD. Similar results were observed for mortality. Obviously, stratification by BD was associated with superior discrimination of trauma patients with respect to outcome and need for early blood products. In this context, ATLS seems to dramatically underestimate the need for blood product transfusion, particularly in group III and IV patients.

In summary, we suggest assessing patients in the ED on the basis of BD. Davis and colleagues [6] have already proposed that, in patients with a BD of less than 6 mmol/L, blood typing should be sufficient but that patients with a BD of at least 6 mmol/L should undergo blood typing and cross-match. Given MT rates and the identification of patients who are in need of emergent transfusion, a BD of 6 mmol/L could also be suggested as a threshold. Table 4 displays our suggestion for a modified version of the current ATLS classification of hypovolemic shock based upon BD as a principal trigger for action. Following the ATLS paradigm of 'keep algorithms simple', specific recommendations are presented with regard to preparation and use of blood products. For class I and II patients, a careful observation should be sufficient unless clinical circumstances dictate otherwise. In class III patients, preparation for transfusion should be initiated. In class IV patients, in whom MT rates were more than 50%, the trauma leader should definitely be prepared for an MT (for example, by activation of an MT protocol and corresponding logistics).

**Table 4:** A new base deficit-based classification of hypovolemic shock

	Class I	Class II	Class III	Class IV
Shock	No shock	Mild	Moderate	Severe

Base deficit at admission, mmol/L	≤ 2	> 2.0 to 6.0	> 6.0 to 10.0	> 10.0

Need for blood products	Watch	Consider	Act	Be prepared for massive transfusion

The retrospective nature of this study and the modifications applied to the ATLS classification in order to conduct the present analysis are clear limitations of this study, and the authors are aware of this shortcoming. Although POCT can provide BD within minutes after ED admission, not every ED is equipped with this technology. However, ATLS claims that the knowledge and skills taught are easily adapted to all venues of trauma care. This implies that every ED worldwide as well as pre-hospital systems (Pre-hospital Trauma Life Support) use similar principles and assessment tools as suggested by ATLS. However, this study may be a first step toward a 'modified ATLS classification of hypovolemic shock' with improved clinical applicability. Further validation on other trauma databases and in prospective studies is needed, especially on cohorts including higher numbers of penetrating injuries. In the absence of POCT, future research is needed to develop alternative approaches (for example, modified and clinically adopted combinations of vital signs), which can be used as an equivalent to BD in the initial assessment of hypovolemic shock. Hereby, the basic and underlying ATLS concept focusing on its intentionally simple applicability, independent of venue, technical prerequisites, and time scales, would be preserved.

## Conclusions

BD upon ED admission indicates the acute presence of hypovolemic shock related to the need for hemostatic resuscitation, transfusion, laboratory findings, and mortality. The four proposed classes of worsening BD seem to predict transfusion requirements and mortality more appropriately than the current ATLS classification of hypovolemic shock. BD might be a relevant clinical approach to early risk-stratify severely injured patients in the state of hypovolemic shock and for blood product transfusion during initial assessment.

## Key messages

• The early recognition and management of hypovolemic shock remain among the most challenging tasks in the initial assessment of trauma patients.

• The current Advanced Trauma Life Support (ATLS) classification of hypovolemic shock displays deficits in reflecting clinical reality; therefore, we propose a new hypovolemic shock classification based on a metabolic marker sensitive to blood loss by measuring tissue perfusion (for example, base deficit (BD)

• A classification based on four groups of worsening BD correlates with the extent of hypovolemic shock in severely injured patients, as reflected by increased transfusion requirements, higher massive transfusion, and mortality rates.

• The new BD-based classification discriminates better the need for early blood product transfusion and mortality in severely injured patients than the current ATLS classification of hypovolemic shock.

## Abbreviations

ATLS: Advanced Trauma Life Support; BD: base deficit; ED: emergency department; GCS: Glasgow Coma Scale; HR: heart rate; IMC: intermediate care; ICU: intensive care unit; MOF: multiple organ failure; MT: massive transfusion; POCT: point-of-care testing; PWH: Prince of Wales Hospital; RISC: revised injury severity classification; SBP: systolic blood pressure; SI: shock index; TARN: Trauma Audit and Research Network; TASH: Trauma-Associated Severe Hemorrhage.

## Competing interests

The authors declare that they have no competing interests. This is an unfunded study.

## Authors' contributions

MMu contributed to study design, acquisition and interpretation of data, and drafting of the manuscript. UN and BB contributed to analysis and interpretation of data and to revision of the manuscript. TB, AW, TF, and TP contributed to study design and to revision of the manuscript. MMae contributed to study conception and design, acquisition of data, analysis and interpretation of data, and revision of the manuscript. All authors read and approved the final manuscript.
